# High Low-Density Lipoprotein Cholesterol Inversely Relates to Dementia in Community-Dwelling Older Adults: The Shanghai Aging Study

**DOI:** 10.3389/fneur.2018.00952

**Published:** 2018-11-12

**Authors:** Fen Zhou, Wei Deng, Ding Ding, Qianhua Zhao, Xiaoniu Liang, Fei Wang, Jianfeng Luo, Li Zheng, Qihao Guo, Zhen Hong

**Affiliations:** ^1^Department of Biostatistics, School of Public Health, Fudan University, Shanghai, China; ^2^Key Lab of Health Technology Assessment, National Health and Family Planning Commission of the People's Republic of China (Fudan University), Shanghai, China; ^3^Key Laboratory of Public Health Safety of Ministry of Education (Fudan University), Shanghai, China; ^4^Institute of Neurology, Huashan Hospital, Fudan University, Shanghai, China; ^5^National Clinical Research Center for Aging and Medicine, Huashan Hospital, Fudan University, Shanghai, China

**Keywords:** low density lipoprotein cholesterol, cognitive impairment, dementia, population-based study, propensity score matching

## Abstract

**Background:** The relationship between cholesterol and cognitive function is unclear from the previous studies. This study was conducted to explore this association in older Chinese adults.

**Methods:** Data were from the Shanghai Aging Study, comprising 3,836 residents aged 50 years or over in an urban community. Diagnoses of dementia and mild cognitive impairment were established according to the fourth edition of diagnostic and statistical manual of mental disorders (DSM-IV) and Petersen criteria. Multivariate logistic regression models, non-matched and propensity score (PS) matched, were used to examine the association between cholesterol levels and cognitive function.

**Results:** There was a significantly higher proportion of participants with low levels of total cholesterol (TC) and low density lipoprotein cholesterol (LDL-C) in the dementia group than in groups without dementia (*P* < 0.05). High LDL-C level was inversely associated with dementia, with a negative trend in the PS matched model. TC and high density lipoprotein cholesterol (HDL-C) were not significantly related to dementia in either non-matched models or PS matched models.

**Conclusion:** Our result indicates that high level of LDL-C is inversely associated with dementia. High level of LDL-C may be considered as a potential protective factor against cognition decline.

## Introduction

Dementia is a progressive brain disorder accompanied by a deterioration in memory and thinking, and often with a decrease in motivation and emotional and language problems. It has physical, mental, economic, and social effects, not only on the patients but also on their careers, their families and society at large. Worldwide, there are over 50 million prevalent dementia cases, and nearly 10 million new cases are diagnosed per year (1). It is estimated that the existing worldwide costs of dementia are US $818 billion in 2017 and that it will become a trillion-dollar disease by 2018—equivalent to the world's 18th largest economy (2).

The brain contains 25–30% of the total body cholesterol. As one of the most essential components of neurons, cholesterol is of great importance to develop and maintain neuronal plasticity and function (3). Studies from western countries have investigated how cholesterol levels related to cognitive impairment, but have yielded inconsistent and conflicting results. In China, only a few studies have investigated the relationship between cholesterol and cognitive impairment among older community residents. The results were varied due to different study design and target population. A cross-sectional study with 597 participants in southwest China has reported that low (TC) (OR = 0.94) and (LDL-C) (OR = 0.94) were associated with mild cognitive impairment (MCI) after adjusting for age, gender and education (4). Another cross-sectional study involving 2,000 community dwellings in eight longevity areas found that higher cholesterol, including TC (OR = 0.73), LDL-C (OR = 0.82), and (HDL-C) (OR = 0.81), was associated with better cognitive function in the oldest old (5). Furthermore, an inversely U-shaped effect of TC on cognition has been observed in a multicenter study with 1,889 people aged 65 years and over from four rural counties (6).

The Shanghai Aging Study is a population-based cohort study with a design, operational procedures and diagnostic criteria similar to most cohort studies in western countries. The study is intending to examine the prevalence and incidence of dementia and MCI as well as their risk and protective factors (7). We analyzed the baseline data of this cohort to explore the association between cholesterol levels and cognitive function among older community dwellers in urban China.

## Methods

### Study population

Between January 2010 and December 2012, 3,836 residents aged ≥50 years were recruited from Jingansi community in central downtown Shanghai, China. Participants were excluded from the study if they were (1) living in nursing homes or other institutions; (2) experiencing mental deficiency or severe schizophrenia, according to their medical record or diagnoses from neurologists; or (3) having severe impairment of hearing, vision, or verbal such that could not accomplish the neuropsychological evaluation. Detailed recruitment procedures were published previously (8).

This study was approved by the Medical Ethics Committee of Huashan Hospital, Fudan University, Shanghai, China (approval number: HIRB2009-195). All participants and/or their legally acceptable representatives provided signed written informed consent to participate this study.

### Characteristics and medical history

Demographic and lifestyle characteristics were collected via a face-to-face questionnaire survey. Data included age, sex, education background, and smoking habits (smoking: regular cigarette smoking more than 1 year). Participants' heights and weights were measured and were used to calculate body mass index (BMI). History of hypertension, diabetes, stroke, and coronary artery disease was collected and confirmed with participants' medical records.

### Laboratory test

A 2-ml blood sample was collected from each participant by research nurses in the morning after 12 h overnight fasting. Blood samples were sent to the central laboratory in Huashan hospital. Cholesterol profiles were measured from serum by Hitachi 7600 full automatic biochemical analyzer. TC was measured with oxidase method and LDL-C and HDL-C were measured with direct method.

### Neuropsychological and neurological assessments

**Figure 1:**
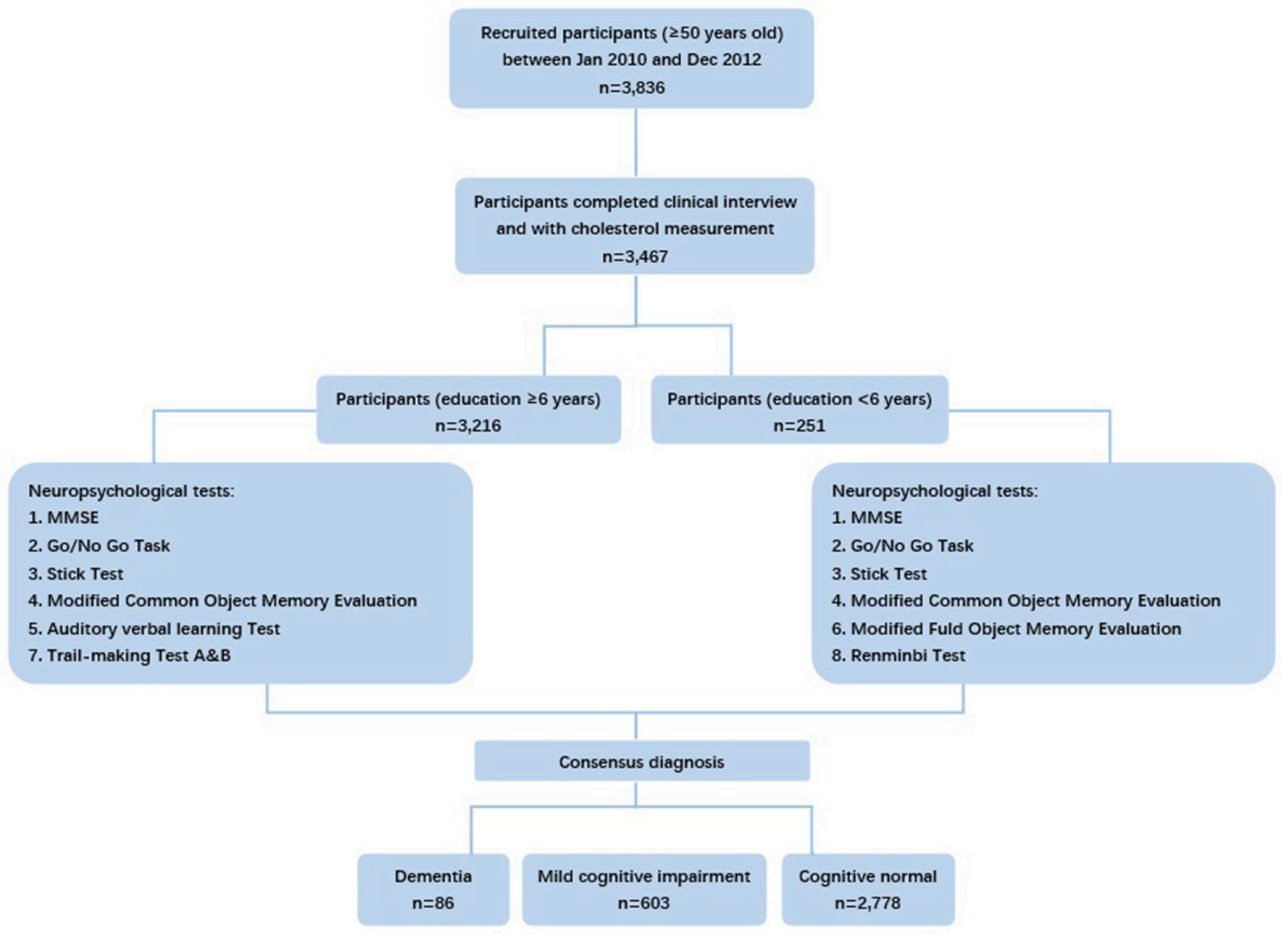
Flowchart of participants' recruitment and deciding neuropsychological tests in our study.

Considering different cultural backgrounds, neuropsychological tests from western countries were translated, adapted and normalized for the Chinese population. Tests applied in our study evaluated global cognition, attention, memory, language, executive function, and spatial construction function of each participant. The tests included: (1) the Mini-Mental State Examination (MMSE); (2) the Conflicting Instructions Task (Go/No Go Task); (3) the Stick Test; (4) the Modified Common Objects Sorting Test; (5) the Auditory Verbal Learning Test; (6) the Modified Fuld Object Memory Evaluation; (7) the Trail-making test A&B; (8) the Renminbi (official currency of China) Test, translated from the EURO test. Participants with at least 6 years of education were given tests 1 to 5 and 7, whereas those with < 6 years of education were given tests 1 to 4 and 6 and 8 (Figure 1). Normative data and more details on these tests were reported elsewhere (8).

The motor responses and reflexes of participants were examined by neurologists. The Center for Epidemiologic Studies Depression and Scale (CESD) (9) was used to evaluate the depression status of participants within the previous week and a major depressive episode was defined as CESD ≥ 16. The Clinical Dementia Rating (CDR) (10) and the Lawton and Brody Activity of Daily Living (ADL) (11) were also conducted to assess the cognitive complaints and the ability to perform activities of daily living and physical self-maintenance.

### Diagnoses of cognitive function

The consensus diagnosis of cognitive function was made by an expert panel (two neurologists, one neuropsychologist, and one neuro-epidemiologist) on the basis of all available information for each participant, including medical, neuropsychological, and neurological data and where possible computed tomography (CT) and magnetic resonance images (MRI) scans. Dementia was diagnosed according to DSM-IV criteria (12). A diagnosis of MCI, referring to Petersen's criteria (13), was considered only for subjects without dementia.

### APOE genotype assessment

Because the APOE-ε4 variant has been found as the largest known genetic risk factor for Alzheimer's disease in a variety of ethnic groups (14), we assessed APOE genotype in our cohort. The extraction of DNA from blood or saliva samples and the assay of APOE genotyping through the TaqmanSNP method (15) were conducted in the central laboratory in Huashan hospital. APOE-ε4 positivity was defined by the occurrence of one or two ε4 alleles.

### Statistical analysis

TC, LDL-C, and HDL-C values were divided into three levels according to tertiles (5): low TC level, < 4.87 mmol/L; moderate TC level, 4.87–5.72 mmol/L; high TC level, >5.72 mmol/L; low LDL-C level, < 2.9 mmol/L; moderate LDL-C level, 2.9–3.7 mmol/L; high LDL-C level, >3.7 mmol/L; low HDL-C level, < 1.17 mmol/L; moderate HDL-C level, 1.17–1.45 mmol/L; and high HDL-C level, >1.45 mmol/L. Differences in characteristics among groups with different cognition status (normal, MCI, or dementia) were evaluated with Kruskal–Wallis test for continuous variables and Cochran–Mantel–Haenszel statistics for categorical variables (16, 17).

Multivariate logistic regression models (non-matched models) were used to examine the association between cholesterol levels and cognition status (MCI or dementia compared with normal). TC, LDL-C, and HDL levels were included in the logistic model as dummy variables, with the low level group used as the reference to estimate the odds ratio (OR) and 95% confidence interval (CI), and with adjustment for covariates such as age, sex, years of education, BMI, depression, diabetes, stroke, hypertension, and APOE-ε4 genotype.

PS methods are being used to reduce the impact of treatment selection bias in the estimation of causal treatment effects using observational data (18). They also can be used to estimate the relationship between any non-randomized factors (19). The PS is the estimated conditional probability of assignment to a particular “treatment” given a series of vector covariates (20). In our study, PS matching was applied to reduce the bias due to non-random exposures (cholesterol levels) and potential confounders; the conditional probability of assignment to moderate levels of cholesterol was chosen as the PS. Matching was conducted in a stepwise fashion. First, we matched the PS in the low cholesterol group with those in the moderate cholesterol group, with 0.05 as a caliper (upper limit of the allowed difference in the scores). Subsequently, the scores in the high cholesterol group were matched to the effective matched pair from the first step, and the mean difference between the high cholesterol group and the other two groups was also not allowed to exceed 0.05. Finally, we obtained matched subgroups with a more balanced distribution of covariates. Multivariate logistic regression models (PS matched models) were then performed by using the PS matched subgroups in a sensitivity analysis, with adjustment of the same covariates as in non-matched models.

All analyses were conducted by SAS 9.4 (SAS Institute Inc., Cary, NC, United States). *P* < 0.05 (two-tailed) were considered statistically significant.

## Results

The full analysis set of this study comprised 3,467 participants who had completed diagnosis of cognition and measurement of TC, LDL-C, and HDL-C (Figure 1). As shown in Table 1, the analyzed participants were characterized by old age (mean [SD] = 69.4 [8.0] years old) and relatively high level of education (mean [SD] = 11.7 [4.0] years). Among them, 86 were diagnosed as dementia and 603 were diagnosed as MCI. The characteristics of age, education, MMSE, history of stroke, hypertension, diabetes, depression, and APOE-ε4 allele carrier status were significantly different among the normal, MCI, and dementia groups.

**Table 1:** Characteristics and cholesterol profiles in 3,467 Chinese adults with different diagnoses of cognition at baseline.

	**Total (*N* = 3,467)**	**Normal (*N* = 2,778)**	**MCI (*N* = 603)**	**Dementia (*N* = 86)**	***P* value^#^**
Sex, female, n (%)	1,893 (54.6)	1,511 (54.4)	331 (54.9)	51 (59.3)	0.4772
Age, year, mean (SD)	69.4 (8.0)	68.5 (7.5)	72.4 (8.7)	78.2 (7.7)	< 0.0001^**^
BMI, mean (SD)	24.3 (3.4)	24.3 (3.4)	24.4 (3.6)	24.0 (3.9)	0.5424
Education, year, mean (SD)	11.7 (4.0)	12.2 (3.7)	10.1 (4.6)	8.0 (6.0)	< 0.0001^**^
Smoking, n (%)	377 (10.9)	297 (10.7)	75 (12.5)	5 (5.8)	0.9500
Stroke, n (%)	377 (10.9)	275 (9.9)	87 (14.5)	15 (17.7)	0.0001^**^
Hypertension, n (%)	1,769 (51.2)	1,370 (49.4)	339 (56.6)	60 (70.6)	< 0.0001^**^
Diabetes, n (%)	460 (13.3)	344 (12.4)	104 (17.4)	12 (14.1)	0.0064^**^
APOE-ε4 (+), n (%)	589 (17.7)	456 (17.0)	114 (20.0)	19 (22.6)	0.0349^*^
Depression, n (%)	599 (17.3)	439 (15.9)	135 (22.5)	25 (29.1)	< 0.0001^**^
ADL, mean (SD)	20.7 (4.2)	20.3 (2.3)	21.1 (4.7)	32.0 (15.8)	< 0.0001^**^
MMSE, mean (SD)	28.0 (2.7)	28.6 (1.6)	26.8 (2.7)	16.8 (5.5)	< 0.0001^**^
TC, mmol/L, mean (SD)	5.4 (1.0)	5.4 (1.1)	5.3 (1.0)	5.2 (1.0)	0.1153
LDL-C, mmol/L, mean (SD)	3.3 (0.9)	3.3 (0.9)	3.3 (0.9)	3.2 (1.0)	0.0921
HDL-C, mmol/L, mean (SD)	1.3 (0.3)	1.3 (0.3)	1.3 (0.4)	1.4 (0.3)	0.6733
TC, category					0.0290^*^
Low	1,148 (33.1)	902 (32.5)	211 (35.0)	35 (40.7)
Moderate	1,169 (33.7)	935 (33.7)	206 (34.2)	28 (32.6)
High	1,150 (33.2)	941 (33.9)	186 (30.9)	23 (26.7)
LDL-C, category					0.0221^*^
low	1,073 (31.0)	843 (30.4)	193 (32.0)	37 (43.0)
Moderate	1,307 (37.7)	1,047 (37.7)	231 (38.3)	29 (33.7)
High	1,083 (31.3)	884 (31.9)	179 (29.7)	20 (23.3)
HDL-C, category					0.5419
Low	1,148 (33.2)	918 (33.1)	203 (33.7)	27 (31.4)
Moderate	1,175 (33.9)	951 (34.3)	199 (33.0)	25 (29.1)
High	1,140 (32.9)	905 (32.6)	201 (33.3)	34 (39.5)

# *Comparison among groups with different diagnoses of cognition*.

* *P-value < 0.05*,

** *P-value < 0.01*.

*MCI, mild cognition impairment; BMI, body mass index; MMSE, Mini-Mental State Examination; APOE-ε4 (+), ApoE-ε4 allele positive; ADL, Activities of Daily Living; TC, total cholesterol; LDL-C, low-density lipoprotein cholesterol; HDL-C, high-density lipoprotein cholesterol*.

*TC: low, < 4.87 mmol/L; moderate, 4.87–5.72 mmol/L; high, >5.72 mmol/L*.

*LDL-C: low, < 2.9 mmol/L; moderate, 2.9–3.7 mmol/L; high, >3.7 mmol/L*.

*HDL-C: low, < 1.17 mmol/L; moderate, 1.17–1.45 mmol/L; high, >1.45 mmol/L*.

Significant differences in the category distributions of TC and LDL-C were found in groups with different cognition status. The lowest proportion was found in participants with dementia who had high level of TC (26.7%) and LDL-C (23.3%).

After adjusting for age, sex, years of education, APOE-ε4 genotype, BMI, depression, diabetes, hypertension and stroke, the non-matched models indicated that the OR of high LDL-C level (OR [95%CI] = 0.56[0.30, 1.04]) had an upper limit awfully close to the null threshold. A U-shaped relationship between HDL-C levels and ORs was found (P for trend = 0.0084) despite of the non-significant effect of HDL-C on dementia when the low level acted as the reference (Table 2). The effects of all the covariates adjusted were showed in the Supplementary Material.

**Table 2:** Odds ratios for TC, LDL-C and HDL-C among participants with dementia vs. normal, and with mild cognitive impairment vs. normal, by non-matched and PS matched models.

	**Non-matched Models**						**PS Matched Models**					
	**MCI vs. Normal**			**Dementia vs. Normal**			**MCI vs. Normal**			**Dementia vs. Normal**		
	***n***	**OR (95%CI)**	**P for trend**	***n***	**OR (95%CI)**	**P for trend**	***n***	**OR (95%CI)**	**P for trend**	***n***	**OR (95%CI)**	**P for trend**
TC			0.6118			0.2349			0.7690			0.1290
Low	1,063	1.00 (reference)		899	1.00 (reference)		720	1.00 (reference)		612	1.00 (reference)
Moderate	1,085	1.00 (0.79, 1.26)		919	0.76 (0.42, 1.35)		730	1.06 (0.80, 1.41)		610	0.76 (0.38, 1.54)
High	1,076	0.95 (0.74, 1.21)		923	0.69 (0.37, 1.27)		728	0.96 (0.73, 1.28)		615	0.56 (0.27, 1.19)
LDL-C			0.3684			0.5047			0.9439			0.0405*
Low	991	1.00 (reference)		845	1.00 (reference)		815	1.00 (reference)		704	1.00 (reference)
Moderate	1,215	1.02 (0.81, 1.28)		1,026	0.67 (0.39, 1.18)		825	1.00 (0.77, 1.31)		699	0.61 (0.32, 1.15)
High	1,018	0.99 (0.77, 1.26)		870	0.56 (0.30, 1.04)		830	0.99 (0.76, 1.30)		706	0.50 (0.26, 0.98)
HDL-C			0.2196			0.0084**			0.7776			0.5566
Low	1,070	1.00 (reference)		909	1.00 (reference)		666	1.00 (reference)		575	1.00 (reference)
Moderate	1,106	1.06 (0.84, 1.34)		938	0.89 (0.47, 1.66)		678	1.14 (0.84, 1.53)		574	0.81 (0.36, 1.82)
High	1,048	1.06 (0.82, 1.36)		894	1.18 (0.64, 2.18)		661	1.05 (0.78, 1.43)		572	1.21 (0.59, 2.47)

*Non-matched models and PS matched models adjusted for age, sex, years of education, ApoE-ε4 allele carrier status, BMI, depression, diabetes mellitus, hypertension and stroke*.

* P-value < 0.05, **P-value < 0.01.

*TC: low, < 4.87 mmol/L; moderate, 4.87–5.72 mmol/L; high, >5.72 mmol/L*.

*LDL-C: low, < 2.9 mmol/L; moderate, 2.9–3.7 mmol/L; high, >3.7 mmol/L*.

*HDL-C: low, < 1.17 mmol/L; moderate, 1.17–1.45 mmol/L; high, >1.45 mmol/L*.

The inverse associations were significant between high LDL-C level and dementia, whereas the associations between TC or HDL-C levels and dementia remained non-significant, according to the PS matched models (Table 2). In addition, the effect of LDL-C was higher than that in the non-matched model. Compared with those with low level, participants with high level of LDL-C were less likely to have dementia (OR [95%CI] = 0.50[0.26, 0.98]) and the OR reduced with the increasing levels (P for trend = 0.0405).

## Discussion

In this community-based cross-sectional study, high level of LDL-C was found to be inversely associated with dementia in older Chinese adults, after controlling for demographic characteristics, health behavior, mood assessment, and the medical history of participants.

Our findings are consistent with those from several prior studies. Higher LDL-C (OR [95%CI] = 0.82[0.70, 0.96]) was reported to be significantly related with higher MMSE scores among the oldest old (aged 80+years) in a community-based cross-sectional study with 2,000 subjects from eight longevity areas in China, after adjusting for age, gender, residence, marital status, education level, current alcohol drinking habits, current cigarette smoking practices, sleep quality, anemia, central obesity, hypertension, diabetes, and chronic kidney disease (5). Another cross-sectional study in South Korea did not find a significant association between (TC) levels (non-fasting) and cognition function in Alzheimer's disease groups (21). Two additional cross-sectional studies in Chinese population also reported a non-significant relationship (6, 22). However, there are also some studies with different findings. For example, elevated level of TC (≥225.01 mg/dl: OR [95%CI] = 1.77 [1.03, 3.04]) was reported to be a risk factor for vascular dementia in a cross-sectional analysis among 2,820 Medicare recipients aged 65+ years in New York (23). Moreover, higher LDL-C level was found to be associated with higher amyloid deposition in a medical center-based study with 74 elderly subjects aged 78 years on average (24).

Elevated HDL-C was found to be negatively associated with cognitive impairment (lower HDL-C: OR [95%CI] = 1.45[1.12, 1.88]) in the Korean Urban Rural Elderly (KURE) study, which involved 3,514 adults aged 65+ years (25). In the Maine-Syracuse study with 540 participants aged 60+ years and without dementia and stroke, a positive relationship between HDL-C and cognitive performance was reported (MMSE: β = 0.195, *P* = 0.006) (26). The PAQUID study, involving 334 French elderly subjects aged 73+ years, also had a similar finding (higher HDL-C: OR [95%CI] = 0.10[0.02, 0.53]) (27). A study among 130 Australian women with mean age of 62.5 years found a much earlier positive effect of HDL-C (non-fasting) on verbal memory (28). Another study in the Czech Republic, involving 141 adults (MMSE score≥24, 69 years of age on average, 47% female, 14.4 years of education on average) found an association between HDL-C and better composite cognitive scores (β = 0.30, *P* = 0.026), only among women (29). Our study, however, did not find a significant association between HDL-C and cognitive status.

Although the exact biological mechanism of LDL-C's potential protective effect is still unknown, we conjecture that one possibility is that high LDL-C might indicate a good nutritional status or health condition. Lower cholesterol has been found to be associated with a higher mortality in the elderly (30, 31), and it may accompany malnutrition, chronic diseases and cancer (32, 33), which in turn may positively associate with cognitive decline (34). On the other hand, because cholesterol is a major component of the brain, it is possible that decreasing cholesterol levels in the elderly is associated with cerebral atrophy, a typical anatomic syndrome of dementia (3). Another speculation is that high LDL-C could reduce neurons' impairments or facilitate compensatory repair of injured neurons (35). The inhibitions of dendrite outgrowth (36) and synaptogenesis (37), and the acceleration of neurodegeneration (38) have been observed when neurons was a short of cellular cholesterol or cholesterol supply. Besides, cholesterol plays an important role in the synthesis, transportation and metabolism of steroid hormones as well as lipid-soluble vitamins, both of which have an impact on synaptic integrity and neurotransmission (37, 39, 40). Apart from the reasons mentioned above, there is another hypothesis about selective survival: participants aged 70+ are more likely to be resistant to the adverse effects of high LDL-C or other cerebrovascular risk factors (41). For example, oldest-old individuals were found to show more variability in cognitive function (42) and to be less apt to age-related cognitive decline (43). At the same time, these participants may also be less susceptible to the benefit of high HDL-C. Further, the protective effect of HDL-C is inconsistent. Indeed, some genetic mechanisms raising plasma HDL-C did not lower risk of myocardial infraction (44) and even the Tromsø study reported that high HDL-C could increase the future risk of venous thromboembolism in female subjects (45).

There might be a possibility of a reversal causality of lipid level and dementia. Participants with dementia may be more likely to have an eating disorder, be malnourished and thus probably accompany with reduction of cholesterol level. However, the cross-sectional study design limited our ability to explore the causal effects. Further prospective studies are needed to provide the evidence to the causality. The second limitation in our study is that we could not discriminate the vascular dementia and Alzheimer's disease because not every participant's MRI was available at baseline. Third, the “snapshot” lipid profile measurements might not be good representative of the actual lipid levels. Fourth, compared with other studies in China, we found that the distribution of cholesterol data and the cut-off values of cholesterol are different with those in the previous Chinese studies, due to the differences in demographic characteristics, regions and socioeconomic status. To be specific, the population of the study by Yue-Bin et al. was in rural areas and characterized by older age (mean age = 85.8 years) and lower education; however, our population was in a high-income city, Shanghai, and characterized by relatively younger age (mean age = 69.4 years), higher education and better economic status. In this regard, the cut-off values in different studies might be less comparable. Further multi-centered population-based studies with larger sample size are needed to verify the U-shape curve of lipid profile. Finally, our findings may not be largely generalizable because our population was characterized by relatively higher education and better economic status than those found in other areas of China; conversely, our findings may be more comparable with that in western studies.

## Conclusion

Our data indicate that high level of LDL-C is inversely associated with dementia in older Chinese adults. High level of LDL-C may be considered as a potential protective factor against cognitive decline. Further long-term prospective studies with a larger sample size and accurate lipid measurement should be carried out to verify this association and to explore underlying pathological mechanisms.

## Author contributions

This work was conceptualized by ZH and DD and all approved the protocol. Data collection was done by DD, QZ, QG, XL, and LZ. Statistical analysis was undertaken by FZ, WD, FW and JL. FZ, WD, and DD prepared the manuscript. WD and DD are the guarantors of this paper.

### Conflict of interest statement

The authors declare that the research was conducted in the absence of any commercial or financial relationships that could be construed as a potential conflict of interest.
