# Supplementary material for: High Low-Density Lipoprotein Cholesterol Inversely Relates to Dementia in Community-Dwelling Older Adults: The Shanghai Aging Study
Source: Front Neurol. 2018 Nov 12;9:952. doi: 10.3389/fneur.2018.00952 (PMC6240682; doi:10.3389/fneur.2018.00952)
Supplement: Supplementary file 1 [file Data_Sheet_1.docx]

**Supplementary Tables**

Table S.1. Odds ratios for potential confounders (when analyzing the effect of TC) among the participants with dementia *vs.* normal, and with mild cognitive impairment *vs.* normal, in the unmatched population.

|  | Unmatched Population | | | | |
| --- | --- | --- | --- | --- | --- |
|  | MCI *vs.* Normal | |  | Dementia *vs.* Normal | |
|  | OR | P value |  | OR | P value |
| TC |  |  |  |  |  |
| Low | ref. | - |  | ref. | - |
| Moderate | 0.996 | 0.9761 |  | 0.756 | 0.3449 |
| High | 0.947 | 0.6602 |  | 0.690 | 0.2356 |
| Age | 1.053 | <.0001 |  | 1.152 | <.0001 |
| Years of Education | 0.889 | <.0001 |  | 0.839 | <.0001 |
| BMI | 0.985 | 0.2974 |  | 0.936 | 0.0764 |
| Sex |  |  |  |  |  |
| Male | ref. | - |  | ref. | - |
| Female | 0.812 | 0.0484 |  | 0.991 | 0.9748 |
| Depression |  |  |  |  |  |
| No | ref. | - |  | ref. | - |
| Yes | 1.485 | 0.001 |  | 1.526 | 0.1472 |
| APOE4 |  |  |  |  |  |
| No | ref. | - |  | ref. | - |
| Yes | 1.261 | 0.0604 |  | 1.963 | 0.0208 |
| Diabetes |  |  |  |  |  |
| No | ref. | - |  | ref. | - |
| Yes | 1.192 | 0.1978 |  | 0.846 | 0.6406 |
| Hypertension |  |  |  |  |  |
| No | ref. | - |  | ref. | - |
| Yes | 1.095 | 0.379 |  | 1.822 | 0.031 |
| Stroke |  |  |  |  |  |
| No | ref. | - |  | ref. | - |
| Yes | 1.029 | 0.8487 |  | 0.901 | 0.7576 |

Table S.2. Odds ratios for potential confounders (when analyzing the effect of LDL) among the participants with dementia *vs.* normal, and with mild cognitive impairment *vs.* normal, in the unmatched population.

|  | Unmatched Population | | | | |
| --- | --- | --- | --- | --- | --- |
|  | MCI *vs.* Normal | |  | Dementia *vs.* Normal | |
|  | OR | P value |  | OR | P value |
| LDL |  |  |  |  |  |
| Low | ref. | - |  | ref. | - |
| Moderate | 1.015 | 0.8962 |  | 0.673 | 0.1658 |
| High | 0.988 | 0.9265 |  | 0.555 | 0.0667 |
| Age | 1.053 | <.0001 |  | 1.151 | <.0001 |
| Years of Education | 0.889 | <.0001 |  | 0.839 | <.0001 |
| BMI | 0.985 | 0.3003 |  | 0.939 | 0.0885 |
| Sex |  |  |  |  |  |
| Male | ref. | - |  | ref. | - |
| Female | 0.803 | 0.0328 |  | 0.982 | 0.946 |
| Depression |  |  |  |  |  |
| No | ref. | - |  | ref. | - |
| Yes | 1.483 | 0.0011 |  | 1.543 | 0.1376 |
| APOE4 |  |  |  |  |  |
| No | ref. | - |  | ref. | - |
| Yes | 1.260 | 0.0609 |  | 1.99 | 0.0186 |
| Diabetes |  |  |  |  |  |
| No | ref. | - |  | ref. | - |
| Yes | 1.194 | 0.1944 |  | 0.843 | 0.6343 |
| Hypertension |  |  |  |  |  |
| No | ref. | - |  | ref. | - |
| Yes | 1.094 | 0.3824 |  | 1.827 | 0.0302 |
| Stroke |  |  |  |  |  |
| No | ref. | - |  | ref. | - |
| Yes | 1.031 | 0.8379 |  | 0.905 | 0.7658 |

Table S.3. Odds ratios for potential confounders (when analyzing the effect of HDL) among the participants with dementia *vs.* normal, and with mild cognitive impairment *vs.* normal, in the unmatched population.

|  | Unmatched Population | | | | |
| --- | --- | --- | --- | --- | --- |
|  | MCI *vs.* Normal | |  | Dementia *vs.* Normal | |
|  | OR | P value |  | OR | P value |
| HDL |  |  |  |  |  |
| Low | ref. | - |  | ref. | - |
| Moderate | 1.057 | 0.6473 |  | 0.886 | 0.7049 |
| High | 1.055 | 0.6745 |  | 1.181 | 0.5949 |
| Age | 1.053 | <.0001 |  | 1.150 | <.0001 |
| Years of Education | 0.888 | <.0001 |  | 0.836 | <.0001 |
| BMI | 0.986 | 0.3457 |  | 0.943 | 0.1244 |
| Sex |  |  |  |  |  |
| Male | ref. | - |  | ref. | - |
| Female | 0.793 | 0.0277 |  | 0.873 | 0.6102 |
| Depression |  |  |  |  |  |
| No | ref. | - |  | ref. | - |
| Yes | 1.479 | 0.0012 |  | 1.519 | 0.1507 |
| APOE4 |  |  |  |  |  |
| No | ref. | - |  | ref. | - |
| Yes | 1.262 | 0.0585 |  | 1.943 | 0.0225 |
| Diabetes |  |  |  |  |  |
| No | ref. | - |  | ref. | - |
| Yes | 1.202 | 0.1788 |  | 0.853 | 0.6598 |
| Hypertension |  |  |  |  |  |
| No | ref. | - |  | ref. | - |
| Yes | 1.097 | 0.3699 |  | 1.836 | 0.0292 |
| Stroke |  |  |  |  |  |
| No | ref. | - |  | ref. | - |
| Yes | 1.031 | 0.8368 |  | 0.905 | 0.7664 |
